# Fault Diagnosis of Demountable Disk-Drum Aero-Engine Rotor Using Customized Multiwavelet Method

**DOI:** 10.3390/s151026997

**Published:** 2015-10-23

**Authors:** Jinglong Chen, Yu Wang, Zhengjia He, Xiaodong Wang

**Affiliations:** 1 State Key Laboratory for Manufacturing and Systems Engineering, Xi’an Jiaotong University, Xi’an 710049, China; E-Mails: jlstrive2008@mail.xjtu.edu.cn (J.C.); hzjvip123@163.com (Z.H.); 2 Technology Center, CNPC Logging CO., LTD, Xi’an 710077, China; E-Mail: bingxinyunying@163.com

**Keywords:** fault diagnosis, vibration signal analysis, customized ensemble multiwavelet, aero-engine rotor

## Abstract

The demountable disk-drum aero-engine rotor is an important piece of equipment that greatly impacts the safe operation of aircraft. However, assembly looseness or crack fault has led to several unscheduled breakdowns and serious accidents. Thus, condition monitoring and fault diagnosis technique are required for identifying abnormal conditions. Customized ensemble multiwavelet method for aero-engine rotor condition identification, using measured vibration data, is developed in this paper. First, customized multiwavelet basis function with strong adaptivity is constructed via symmetric multiwavelet lifting scheme. Then vibration signal is processed by customized ensemble multiwavelet transform. Next, normalized information entropy of multiwavelet decomposition coefficients is computed to directly reflect and evaluate the condition. The proposed approach is first applied to fault detection of an experimental aero-engine rotor. Finally, the proposed approach is used in an engineering application, where it successfully identified the crack fault of a demountable disk-drum aero-engine rotor. The results show that the proposed method possesses excellent performance in fault detection of aero-engine rotor. Moreover, the robustness of the multiwavelet method against noise is also tested and verified by simulation and field experiments.

## 1. Introduction

Currently, aircrafts have an increasing number of applications in the transportation industry, as well as elsewhere. Furthermore, the safe operation of aircraft is always an important research interest. As one of the most important components of an aircraft, the condition of the aero-engine greatly impacts on the safe operation of the aircraft. Assembly looseness in the aero-engine rotor leads to local stress beyond safe limits on the pressure holes, which leads to a rupture accident of the labyrinth seal toothed disk in the high pressure compressor rotor of an aero-engine, or to catastrophic accidents directly [1]. Thus, fault diagnosis is a significant requirement when scheduling proper maintenance and for avoiding serious accidents. Thus far, many aircrafts have been equipped with condition monitoring and fault diagnosis systems. Vibration analysis might continue to be the one of the most popular and useful approaches employed in the fault detection of mechanical equipment [2,3]. The corresponding important problem of fault detection is how to process the vibration data in order to get the diagnostic feature information. One solution is to describe the vibration data as a process that can be parameterized based on simple statistical analysis (mean, minimum, maximum) or advanced higher order statistics (kurtosis, *etc.*) [4]. One more implementing method is to express the vibration signal in the frequency domain (fast-Fourier transform, *etc.*) [4]. However, this method would make no sense when the vibration data are acquired under a non-stationary operation regime. However, unfortunately, a great number of engineering practices indicate that vibration data gathered from machine-integrated sensors always appears non-stationary. This is why a rupture accident of the labyrinth seal toothed disk in the high pressure compressor rotor still often occurs in many different types of aircrafts, even if they have been equipped with condition monitoring and fault diagnosis systems [1]. Thus, an advanced and effective method should be developed and studied so that it may be introduced for this task.

Many vibration signal-processing methods have been developed for mechanical equipment fault detection, such as envelope analysis method [5], empirical mode decomposition (EMD) [6], stochastic resonance (SR) [7] and spectral kurtosis (SK) [8]. Furthermore, these methods have also been applied in fault detection of mechanical equipment in recent years. However, all these signal processing method have some special limitations relevant to this task. Envelope analysis can effectively detect and extract periodic features, but it also suffers great difficulties in detecting transient features. One of the most typical features of EMD is that intrinsic mode functions (IMFs) are computed on the basis of the cubic spline function. Thus, EMD is sensitive to detect the harmonic feature on the vibration signal but invalid for transient features. SR will be helpless in this type of vibration signal because the signal-to-noise (SNR) ratio is too low. By computing the kurtosis value at “each frequency line”, SK can effectively detect the presence of the hidden non-stationarities in the vibration signal. Thus, SK is ideally suitable for vibration signal processing in long records and possesses special properties: frequency-concentration and sensitive to kurtosis. Therefore, mechanical equipment fault detection still calls for a more effective vibration signal processing method.

As a powerful tool for describing the non-stationary signal, wavelet transform (WT) [9,10] has already shown its tremendous effectiveness in mechanical equipment condition monitoring and fault diagnosis because of its property of multi-resolution analysis [11,12,13]. Different from Fourier transform, a specific fault symptom can be matched and extracted by WT on the basis of selecting the basis function from the basis function library, which is greatly beneficial to fault feature extraction. However, the selection of basis function is not uncontrolled because there are limited basis functions in the library. Furthermore, any inappropriate wavelet basis function employed in the special engineering application will directly lower the accuracy of the condition monitoring and fault diagnosis. Therefore, it is a vital step to select an appropriate wavelet basis function for vibration data processing. In fact, no fixed basis function that is related to the special vibration data can match a data feature entirely in all applications. Moreover, no scalar wavelet in the wavelet basis function library can possess orthogonality, symmetry, compact support and higher order of vanishing moments simultaneously. Unfortunately, these properties are significant for describing vibration data comprehensively and precisely. In addition, multiwavelet transform only pays attention to multi-resolution analysis in low frequency band, which may omit useful condition feature information.

In order to reduce these limitations of the scalar wavelet transform, ensemble multiwavelet analysis method is developed. Multiwavelet transform, as the newer development of the traditional wavelet transform theory, was firstly developed by Geronimo *et al.* [14]. Multiwavelet not only possesses the ability of multi-resolution analysis but also simultaneously grasps such important properties as orthogonality, symmetry, compact support and higher order of vanishing moments that traditional scalar wavelet basis function does not [15]. Since possessing multiple wavelet basis functions, multiwavelet transform does well in identifying signal feature with multiple kinds of shapes for the fault detection. Moreover, ensemble multiwavelet is constructed to avoid a mistake in the selection of the basis functions in a special application. Lifting scheme [16] is a useful method for the wavelet basis function construction developed by Sweldens in recent years. Due to not relying on the Fourier transform and the whole construction step derived in the time domain, it provides much more freedom and flexibility for the construction of biorthogonal wavelet. The performance of any existing wavelet basis function from the basis function library can be enhanced by the lifting scheme according to the actual requirement in the special engineering application, which brings about the possibility of constructing a customized wavelet. Chen *et al.* [13] developed customized lifting multiwavelet based on the common lifting scheme for the condition identification of mechanical equipment. However, the mentioned method has the severe shortcoming of low adaptivity on the developed multiwavelet basis, which limits the ability to customize lifting multiwavelet on signal feature extraction. Wang *et al.* [17] and Chen *et al.* [18] developed customized lifting multiwavelet based on multiwavelet symmetric lifting scheme for the fault detection of mechanical equipment. Ensemble multiwavelet transform is performed to achieve the multi-resolution analysis in the entire signal frequency band, which is beneficial with describing the vibration data precisely and comprehensively. The remaining important problem to solve is how to reflect and evaluate the current state of mechanical equipment by a simple rule based on the vast customized ensemble multiwavelet transform coefficients.

Information entropy was first proposed by Shannon in 1948 as a useful indicator to reflect and evaluate the uncertain degree of a system [19]. Based on the information entropy theory, the largest entropy value means the most uncertain probability distribution and the smallest entropy value equals the most certain probability distribution. From then on, more and more scholars have joined in the study of information entropy for special application. Ren *et al.* [20] proposed the contents of wavelet entropy and relative wavelet entropy to detect structural damage. EI Safty *et al.* [21] developed the wavelet information entropy method with neural-fuzzy inference system for fault detection in transmission lines and to identify the phases related to the fault of power systems. Lin *et al.* [22] identified the misalignment fault of the motor shaft based on multi-scale entropy for wavelet denoising. Many kinds of definitions for information entropy have been developed in detail for some special and concentrated applications, such as fuzzy entropy [23] for customized bacterial foraging, sample entropy [24] for the tool condition detection, hierarchical entropy [25] for biological data analysis, cross entropy [26] for the multi-target tracking, Rényi entropy [13,27] for high-resolution scalar quantization, and so on. Since wavelet transform has the powerful ability to describing un-stationary vibration data in both the time and frequency domains. The definition of wavelet information entropy is developed to describe the dynamic characteristics of the measured vibration data from mechanical equipment. Based on this, a new method based on the ensemble multiwavelet transform and normalized information entropy is studied for the condition identification of aero-engine rotor.

In this paper, a fault detection method based on ensemble multiwavelet transform is proposed for assembly looseness fault detection in aero-engine rotors. Benefiting from characteristics of multi-resolution analysis and the multiple basis functions, multiwavelet has great advantage when describing non-stationary vibration data. However, the fixed basis functions of conventional discrete multiwavelet transform, which is not related to vibration data, may decrease the preciseness of fault detection. Moreover, multiwavelet transform does not realize multi-resolution analysis in the high frequency band, which may result in omitting some useful fault feature information. To overcome these limitations, a customized multiwavelet basis function is constructed via symmetric lifting scheme. Then, the measured vibration data from the mechanical equipment is processed by the ensemble multiwavelet transform. The relative energy in a decomposed frequency band of the ensemble multiwavelet transform coefficients means a percentage of the whole signal energy is taken as probability. Normalized information entropy is computed on the basis of the relative energy to reflect the current state of a mechanical system. The proposed method is first applied to the fault detection of an experimental aero-engine rotor. Finally, the proposed approach is used for the engineering application and it successfully identified weak crack fault in a demountable disk-drum aero-engine rotor. The results show that the proposed method possesses excellent performance in fault detection of aero-engine rotor.

The rest of this paper is organized as follows. In Section 2, the theory of multiwavelet is summarized briefly. In Section 3, the multiwavelet symmetric lifting scheme is first described and the customized multiwavelet basis function is constructed based on the symmetric lifting scheme. Then, the proposed method, called ensemble multiwavelet analysis method, is proposed. In Section 4, this method is applied to two cases to demonstrate its usefulness and performance. Conclusions are provided in Section 5.

## 2. Summary of Multiwavelet Transform

Multiwavelet is generated by two or more mother wavelets [28]. Similar to the scalar wavelet transform, the theory of multiwavelet is also based on the concept of multi-resolution analysis (MRA) [28]. Multi-scaling function vector $\Phi ={\left[\phi_{1},\phi_{2,}\cdot \cdot \cdot ,\phi_{r}\right]}^{\text{T}}$ and multiwavelet function vector $\Psi ={\left[\psi_{1},\psi_{2},\cdot \cdot \cdot ,\psi_{r}\right]}^{\text{T}}$ satisfy the following two-scale matrix refinement Equations:

(1) $$\Phi (t)=\sqrt{2}\sum_{k=o}^{M}H_{k}\Phi (2t-k)k\in Z$$

(2) $$\Psi (t)=\sqrt{2}\sum_{k=o}^{M}G_{k}\Phi (2t-k)k\in Z$$

The coefficients $\left\{H_{k}\right\}$ and $\left\{G_{k}\right\}$ are r×r matrices instead of scalars and $\Psi ={\left[\psi_{1},\psi_{2},\cdot \cdot \cdot ,\psi_{r}\right]}^{\text{T}}$ denotes the multiwavelet function corresponding to multi-scaling function Φ. In the frequency domain, Equations (1) and (2) are:

(3) $$\hat{\Phi }(\omega )=H(e^{-i\omega /2})\hat{\Phi }(\frac{\omega }{2})$$

(4) $$\hat{\Psi }(\omega )=G(e^{-i\omega /2})\hat{\Phi }(\frac{\omega }{2})$$

H(ω) and G(ω) are the refinement symbols corresponding to Φ and Ψ. The symbols in *Z*-domain are determined by:

(5) $$H(z)=\frac{1}{2}\sum_{k=0}^{M}H_{k}z^{k}\text{ and }G(z)=\frac{1}{2}\sum_{k=0}^{M}G_{k}z^{k}$$

With the starting vector coefficients $\lambda_{0,0},\cdot \cdot \cdot ,\lambda_{0,2^{j}-1}$, the decomposition step of multiwavelet transform is:

(6) $$\lambda_{j-1,n}=\sum_{k}H_{k-2n}\lambda_{j,k}\text{ and }\gamma_{j-1,n}=\sum_{k}G_{k-2n}\lambda_{j,k}$$

Low frequency coefficients $\lambda_{j-1,n}$ and high frequency coefficients $\gamma_{j-1,n}$ after the decomposition step are vectors of *r*-dimension. The reconstruction step of multiwavelet transform is:

(7) $$\lambda_{j,k}=\sum_{n}{H^{*}}_{k-2n}\lambda_{j-1,n}+\sum_{n}G_{k-2n}^{*}\gamma_{j-1,n}$$

Note that the superscript * means the complex conjugate transpose.

Due to the translations and dilations operations of multi-scaling and multiwavelet vector functions, multiwavelet can seize the vital vibration data processing properties of orthogonality, symmetry, compact support and higher order of vanishing moments simultaneously [15], which has been proven to be impossible for scalar wavelets, except Haar wavelet. Thus, multiwavelet transform can describe any vibration data more precisely and comprehensively because of its multi-input and multi-output system. In addition, due to the matrix-valued filter-bank, two or more input streams are needed in the process of multiwavelet transform. However, the processing vibration data would be one input stream usually and so some kind of pre-processing should be done before the implementation of multiwavelet transform. Correspondingly, a post-processing step is needed after the multiwavelet transform and it must be the inverse process of the pre-processing step. There are many kinds of pre-filters with different properties [29]. It has also been proven that the pre-filter algorithm called oversampling is more beneficial to vibration data feature identification than critically sampling ones [30]. Therefore, oversampling algorithm is selected as the multiwavelet preprocessing operation in these applications.

## 3. Ensemble Multiwavelet Analysis Method

### 3.1. Multiwavelet Lifting Scheme

Sweldens developed alifting scheme that made use of an existing wavelet and scaling functions to generate a new wavelet with prescribed or required properties via transformations in the time domain, which makes it possible to produce an adaptive wavelet. Assuming an initial set of biorthogonal filter operators $\left\{H_{j}^{old},{\tilde{H}}_{j}^{old},G_{j}^{old},{\tilde{G}}_{j}^{old}\right\}$, then a new set of biorthogonal filter operators $\left\{H_{j},{\tilde{H}}_{j},G_{j},{\tilde{G}}_{j}\right\}$ can be generated by conducting the lifting scheme as follows [16]:

(8) $$\begin{array}{l} H_{j}=H_{j}^{old}\\ G_{j}=G_{j}^{old}+S_{j}H_{j}^{old}\\ {\tilde{H}}_{j}={\tilde{H}}_{j}^{old}-S_{j}^{*}{\tilde{G}}_{j}^{old}\\ {\tilde{G}}_{j}={\tilde{G}}_{j}^{old} \end{array}$$

**Figure 1:**
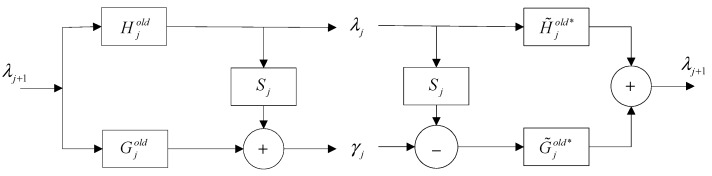
The forward and inverse transform of the lifting scheme.

The forward and corresponding inverse operations of the wavelet lifting scheme are shown in Figure 1. The noteworthy advantage of producing a new wavelet basis function based on the lifting scheme is that the abundant choices of S can be realized. Once S is selected, the lifting scheme ensures and reserves that all filters have the property of biorthogonality. Thus, as can be seen, the foremost advantage of the lifting scheme is that it supplies the possibility of generating a customized wavelet.

Even now, there are remarkable advantages for multiwavelet over scalar wavelet, but the fixed multiwavelet basis function, independent of the special vibration data, is still not the optimal selection for a specified engineering application. Thus, the lifting scheme is used to generate customized multiwavelet basis function or ensemble multiwavelet basis function.

Based on the multiwavelet lifting scheme, a changeable set of biorthogonal filter operators $\left\{H_{j},{\tilde{H}}_{j},G_{j},{\tilde{G}}_{j}\right\}$ can be obtained as follows [18]:

(9) $$\begin{array}{l} H_{new}(z)=H(z)\\ G_{new}(z)=T(z^{2})(G(z)+S(z^{2})H(z))\\ {\tilde{H}}_{new}(z)=\tilde{H}(z)-S^{*}(z^{2})\tilde{G}(z)\\ {\tilde{G}}_{new}(z)={\left(T^{*}(z^{2})\right)}^{-1}\tilde{G}(z) \end{array}$$

where the determinant of T(z) is a monomial and S(z) and T(z) are finite-degree.

One of the most important properties of the multi-scaling function, which has noteworthy significance in engineering applications, is the approximation order. Based on the wavelet theory, we know that if a multi-scaling function owns an approximation order *m*, this indicates that the corresponding multiwavelet function owns *m* vanishing moments. In the following, the procedure of generating a new multiwavelet, based on the multiwavelet lifting scheme, and by use of an original multiwavelet with required numbers of vanishing moments will be explained. Firstly, select the original multiwavelet $\omega_{0}(x)$ $\omega_{0}(x)=\Psi_{1}$ or $\Psi_{2}$) from the basis function library and a set of translation quantity k of scaling functions as well as wavelet functions $\omega_{1}(x),\ldots ,\omega_{k}(x)$. Next, generate the new multiwavelet by use of the “lifting coefficients equation” as follows:

(10) $$\omega_{0}^{new}=\omega_{0}(x)+\sum_{i=1}^{k}c_{i}\omega_{i}(x)$$

If the vanishing moment of a multiwavelet required to be lifted from p to p′, both sides of “lifting coefficients equation” are integrated. Then, a set of linear equations in the matrix form is obtained and displayed as follows:

(11) $$\left[\begin{array}{llll} \int \omega_{1}x^{p}dx & \int \omega_{2}x^{p}dx & \cdots & \int \omega_{k}x^{p}dx\\ \int \omega_{1}x^{p+1}dx & \int \omega_{2}x^{p+1}dx & \cdots & \int \omega_{k}x^{p+1}dx\\ \vdots & \vdots & \ddots & \vdots \\ \int \omega_{1}x^{p\prime -1}dx & \int \omega_{2}x^{p\prime -1}dx & \cdots & \int \omega_{k}x^{p\prime -1}dx \end{array}\right]\left[\begin{array}{c} c_{1}\\ c_{2}\\ \vdots \\ c_{k} \end{array}\right]=\left[\begin{array}{l} -\int \omega_{0}x^{p}dx\\ -\int \omega_{0}x^{p+1}dx\\ \begin{array}{cc} & \vdots \end{array}\\ -\int \omega_{0}x^{p\prime -1}dx \end{array}\right]$$

The solutions $\left\{c_{i}\right\}$ of Equation (11) are exactly the coefficients of functions that are used to perform lifting operation. Equation (10) is carried out to z-transform and the multiwavelet lifting scheme is realized successfully.

### 3.2. Symmetric Lifting Scheme

The vital step in the ensemble multiwavelet transform is the customized construction of the multiwavelet basis function. In this section, the customized multiwavelet basis function is generated on the basis of the symmetric lifting scheme. Symmetry could guarantee the filter has linear phase or generalized linear phase, which is beneficial for perfect reconstruction. However, the symmetry is not realized in the traditional multiwavelet lifting scheme. To realize a symmetric multiwavelet lifting scheme, the vital factor is the appropriate selection of translation quantity k of multiwavelet basis functions [18]. Taking $\Psi_{1}$, for example, and supposing functions $\omega_{i}$ is symmetric or anti-symmetric at points $a_{\omega_{i}}$, respectively. The selection of translation quantity k should meet the following Equation:

(12) $$a_{\psi_{1}}-(a_{\omega_{i}}+k_{\omega_{i},1})=(a_{\omega_{i}}+k_{\psi_{i},2})-a_{\psi_{1}}$$

Note that i=1,  2,  j=1,  2,⋯,  k∈Z.

The symmetry of the original multiwavelet functions and multi-scaling functions can be described as:

(13) $$B_{\omega_{i}}=\pm 1$$

where +1 notes symmetry and −1 notes anti-symmetry. Taking the symmetry conditions into account, Equation (11) turns into the following Equation:

(14) $$\left[\begin{array}{ccc} \int \omega_{1}(x+k_{\omega_{1},1})x^{p}dx & \int \omega_{1}(x+k_{\omega_{1},2})x^{p}dx & \cdots \\ \int \omega_{1}(x+k_{\omega_{1},1})x^{p+1}dx & \int \omega_{1}(x+k_{\omega_{1},2})x^{p+1}dx & \cdots \\ \vdots & \vdots & \vdots \\ \int \omega_{1}(x+k_{\omega_{1},1})x^{p\prime -1}dx & \int \omega_{1}(x+k_{\omega_{1},2})x^{p\prime -1}dx & \cdots \end{array}\right]\left[\begin{array}{ccc} 1 & & 0\\ B_{\omega_{0}}B_{\omega_{1}} & & \\ & \ddots & \\ 0 & & \end{array}\right]\left[\begin{array}{c} c_{1}\\ c_{2}\\ \vdots \\ c_{k} \end{array}\right]=\left[\begin{array}{l} -\int \omega_{0}(x)x^{p}dx\\ -\int \omega_{0}(x)x^{p+1}dx\\ \begin{array}{cc} & \vdots \end{array}\\ -\int \omega_{0}(x)x^{p\prime -1}dx \end{array}\right]$$

The solutions of Equation (14) are the coefficients for the lifting operation $\Psi_{1}$, while the lifting operation on $\Psi_{2}$ is similar to $\Psi_{1}$. Next, substitute the related lifting coefficients into the “lifting coefficients equation”. Then, corresponding lifting matrices *T* and *S* can be obtained through *Z* transform. The direct presentation of the multiwavelet lifting scheme is displayed as follows:

(15) $$G_{new}(z)=T(z^{2})(G(z)+S(z^{2})H(z))$$

A new multiwavelet basis function with the symmetry property is successfully constructed based on the symmetric multiwavelet lifting scheme with the help of T(z) and S(z).

In order to construct ensemble multiwavelet basis function with specified properties, the appropriate free parameters and optimization objective are needed to optimize this process. Equation (14) can be briefly described as *MC* = *N*, where $C={\left[c_{1},c_{2}\cdots ,c_{k}\right]}^{T}$, and matrix *M* means the related coefficient matrix of Equation (14). When the set of Equation (14) is underdetermined, there are N=(p′−p)−Rank(M) free parameters, which mean that ensemble symmetric lifting scheme can be conducted by the optimization of the free parameters.

As described in the Section 1, information entropy is a useful indicator to evaluate the uncertain degree of a system. Thus, choose the minimum entropy principle as an evaluation objective to guide the customized lifting multiwavelet basis function construction. The mathematical definition of the information entropy is displayed as follows [19]:

(16) $$E=-\sum_{i}^{n}p_{i}\ln p_{i}$$

where $\left(p_{1},p_{2},\cdots ,p_{n}\right)$ reflect the probability density functions of the processed data amplitude.

Based on information theory, the uniformity of the probability distribution directly affects the size of the entropy value, and the most certain probability distribution owns the minimum entropy value. Our objective in the customized construction process is to find the optimal multiwavelet basis function for the processed vibration data by searching the minimum value of entropy E.

Obviously, optimization method is an essential tool for searching the optimal parameter. Genetic algorithm [31] on the basis of the idea of natural selection has the enormous advantage that it does not have mathematical requirements on the optimization problem. In addition, a great number of applications have indicated that genetic algorithm is a powerful tool in global optimization. Thus, genetic algorithm is adopted to construct the optimal multiwavelet basis function for a given measured vibration data by selecting the free parameters. According to our experimental experience and to increase the efficiency of the process, the parameters of genetic algorithm are set as follows: the number of iteration is set to 30, the range of the population scale is set to 50, the probability of crossover is set to 0.7 and the probability of mutation is set to 0.05.

### 3.3. Ensemble Multiwavelet Analysis Method

**Figure 2:**
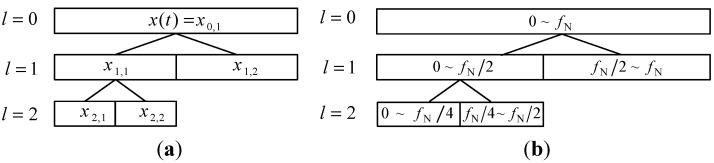
(**a**) The decomposition step of multiwavelet transform and (**b**) the corresponding frequency bands.

**Figure 3:**
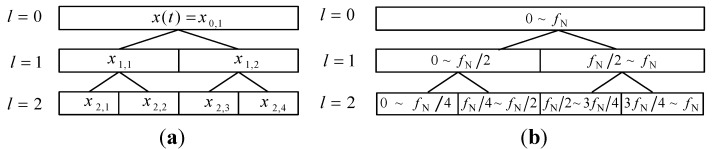
(**a**) The decomposition step of ensemble multiwavelet transform and (**b**) the corresponding frequency bands.

Customized construction of multiwavelet basis function can enhance the ability of multiwavelet on the vibration date feature extraction. However, multiwavelet transform only pays attention to multi-resolution analysis in low frequency band, which may omit the useful abnormal state feature information. The decomposition step of the multiwavelet transform and the corresponding frequency bands are shown in Figure 2. In order to overcome this disadvantage, ensemble multiwavelet transform is performed based on the customized multiwavelet filter-bank. Then, the multi-resolution analysis can be realized in both low frequency band and high frequency band. Thus, ensemble multiwavelet analysis method is a more precise and comprehensive vibration data processing tool than the multiwavelet transform [18,32,33,34]. Let *l* be the transform level and $\left(x_{l,i}\right)$ be the ensemble multiwavelet transform coefficients at the l decomposition in the i frequency band. $f_{N}$ means the upper frequency limit of the signal x(t). Then, the decomposition step of the ensemble multiwavelet transform and the corresponding frequency bands are shown in Figure 3.

The ensemble multiwavelet transform also obeys the energy conservation principle due to its biorthogonal basis. After the l transform, the $2^{l}$ frequency bands can be obtained and each decomposed frequency band owns the same bandwidth. Let $x_{l,i}\left(k\right)$ be the ensemble multiwavelet transform coefficients at the l decomposition in the i frequency band, then its energy $E_{l,i}$ and relative energy ${\tilde{E}}_{l,i}$ are, respectively, calculated as follows:

(17) $$E_{l,i}=\frac{1}{n-1}{\sum_{k=1}^{n}\left(x_{l,i}(k)\right)}^{2},i=1,2,\cdots ,2^{l},k=1,2,\cdots ,n,n\in Z$$

(18) $${\tilde{E}}_{l,i}=E_{l,i}{\left(\sum_{i=1}^{2^{l}}E_{l,i}\right)}^{-1}$$

Obviously, $\sum_{i=1}^{2^{l}}E_{l,i}=1$, the summation of total relative energy equals to 1.

Then, the definition of normalized ensemble multiwavelet information entropy *Ent* can be described as follows:

(19) $$Ent=-\sum_{i=1}^{2^{l}}{\tilde{E}}_{l,i}\log_{2^{l}}{\tilde{E}}_{l,i}$$

In Equation (19), the base of logarithm is $2^{l}$. The value *Ent* belongs to [0, 1] in this definition. We can find that if every frequency band has the same relative energy ${\left(2^{l}\right)}^{-1}$ (like equal probability distribution), then *Ent* = 1; if only one of the $2^{l}$ frequency bands concentrates the whole energy of vibration signal, its relative energy would equal to 1 (like the most certain probability distribution), and then *Ent* = 0. Thus, we can regard *Ent* as normalized information entropy. In this paper, the normalized ensemble multiwavelet transform information entropy is developed and introduced for assembly looseness fault detection in aero-engine rotors.

**Figure 4:**
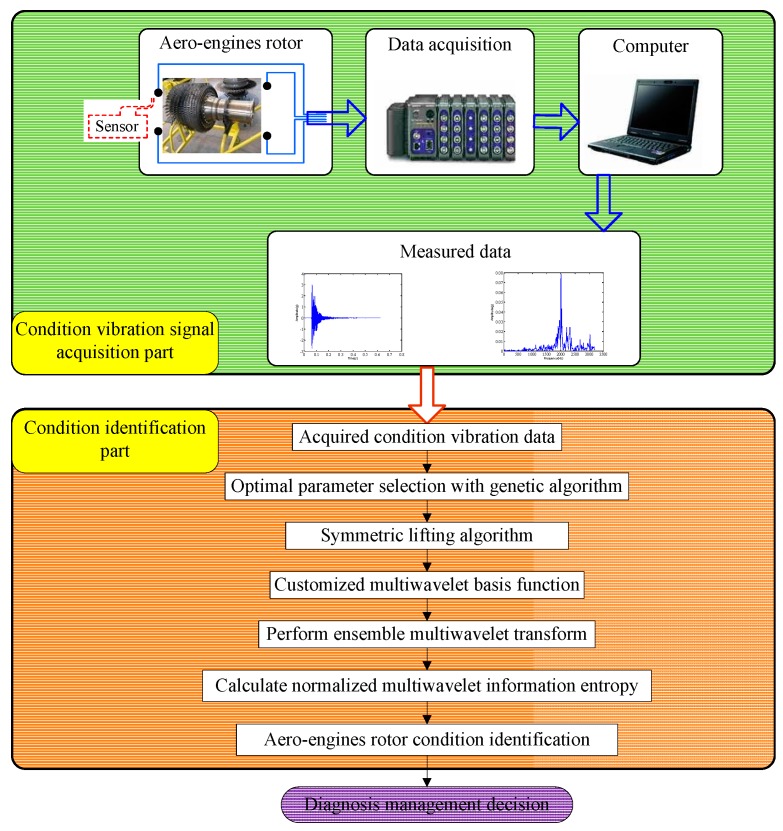
The flow chart of the proposed method.

The procedure of the developed method, called normalized ensemble multiwavelet transform information entropy, can be concluded in the following flow chart, as displayed in Figure 4. Meanwhile, the process of the normalized ensemble multiwavelet transform information entropy for the assembly looseness fault detection of aero-engine rotors can be summarized as follows:

1. Choose the original multiwavelet with the vanishing moment p. Then, with the given vanishing moment p′, the translations of Φ and Ψ are symmetrically selected;
2. Initialize the free parameters $\left\{f_{1},f_{2},\cdots f_{N_{f}}\right\}$ and substitute them into matrix M. Meanwhile, make the matrix satisfy Rank(M)=p′−p;
3. Select the optimal free parameters on the basis of vibration signal by the genetic algorithm and the rule of the minimum information entropy principle;
4. Determine the transform level and conduct the ensemble multiwavelet transform. Obtain the corresponding ensemble multiwavelet transform coefficients;
5. Compute the total energy and the relative energies in each decomposed frequency band. Calculate the normalized information entropy for assembly looseness fault detection in aero-engine rotors.

## 4. Assembly Looseness Fault Identification of Dismountable Disk-Drum Aero-Engine Rotors

In this section, the proposed method is firstly applied to the fault detection of an experimental dismountable disk-drum aero-engine rotor for verifying the performance in this task. Then, the proposed method is used for an engineering application. Further verification is performed by monitoring an aero-engine rotor of the same type, but from the engineering field.

### 4.1. Case 1: Analysis on the Experimental Vibration Signals

**Figure 5:**
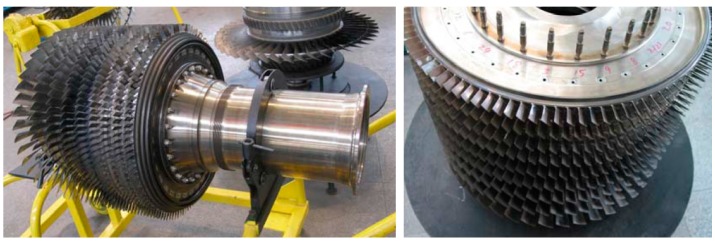
Photograph of the dismountable disk-drum aero-engine rotor.

**Figure 6:**
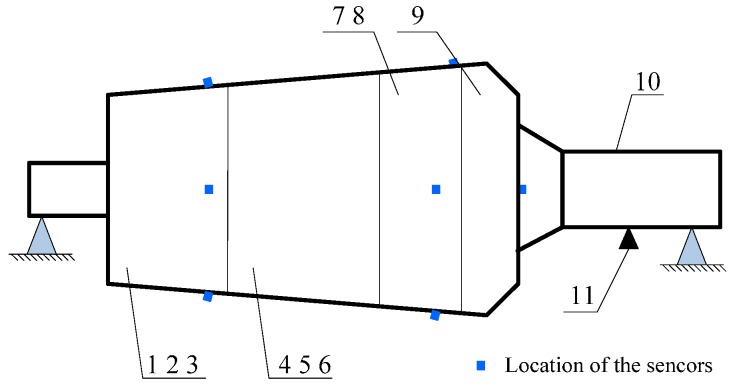
Illustration for structure of the aero-engine rotor and locations of the sensors: 1–9 are the first–ninth disk, 10 is the shaft, 11 is the location of the exciter.

The photograph of the experimental dismountable disk-drum aero-engine rotor is displayed in Figure 5. The experimental system mainly consists of a dismountable disk-drum aero-engine rotor, a data acquisition system, a signal generator, an exciter and sensors. The sketch map of this experimental system is shown in Figure 6. The dismountable disk-drum aero-engine rotor consists of a labyrinth seal toothed disk (serial number 10 in Figure 6), nine disks (serial numbers 1–9 in Figure 6) and a shaft (serial number 11 in Figure 6). An exciter (serial number 11 in Figure 6) is mounted below the shaft to excite the aero-engine rotor. A signal generator is applied to produce excitation vibration signal for the exciter. There are four accelerometers on the sensor installation surface, which is next to the labyrinth seal toothed disk in order to acquire the dynamic response signal of the dismountable disk-drum aero-engine rotor with bolts from looseness to tightness. Sony EX data system acquisition system is adopted to measure and store the dynamic response data.

**Figure 7:**
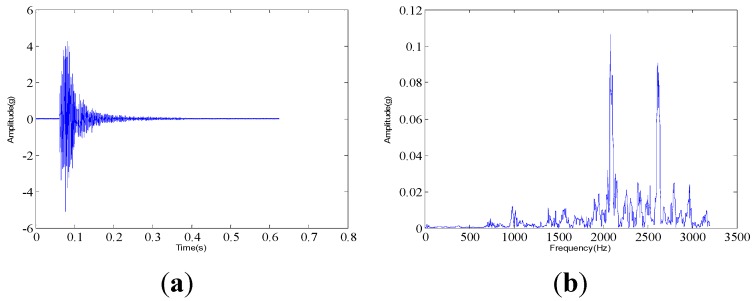
The measured vibration signal under the assembly condition 1 and the corresponding frequency spectrum. (**a**) vibration signal; (**b**) frequency spectrum.

**Figure 8:**
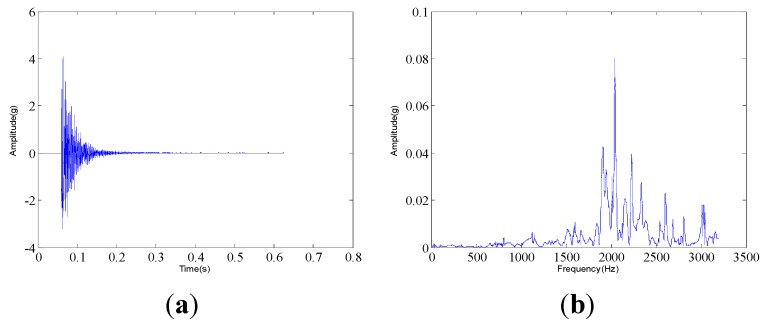
The measured vibration signal under the assembly condition 2 and the corresponding frequency spectrum. (**a**) vibration signal; (**b**) frequency spectrum.

**Figure 9:**
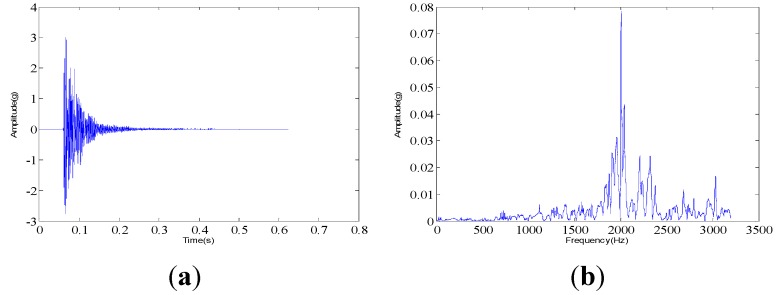
The measured vibration signal under the assembly condition 3 and the corresponding frequency spectrum. (**a**) vibration signal; (**b**) frequency spectrum.

The measured dynamic response data are collected from the experimental system under three different assembled quality conditions: looseness condition 1 (tighten up the dismountable disk-drum aero-engine rotor with force moment M1); looseness condition 2 (tighten up the dismountable disk-drum aero-engine rotor with force moment M2 after condition 1); qualified condition 3 (tighten up the dismountable disk-drum aero-engine rotor with force moment M3 after condition 2), such that M1 < M2 < M3. Four vibration signals are measured by the sensors described above for each condition. Therefore, twelve vibration data are measured under all the three states. The sample frequency is set at 6400 Hz. The measured dynamic response data under each assembled quality state from the same sensor in time domain and the corresponding frequency spectrum are displayed in Figure 7, Figure 8 and Figure 9. The dynamic response signals under each assembled quality condition are damped in the time domain. It is shown that there are no evident features to identify the three different assembled quality conditions directly in the time or frequency domains.

**Figure 10:**
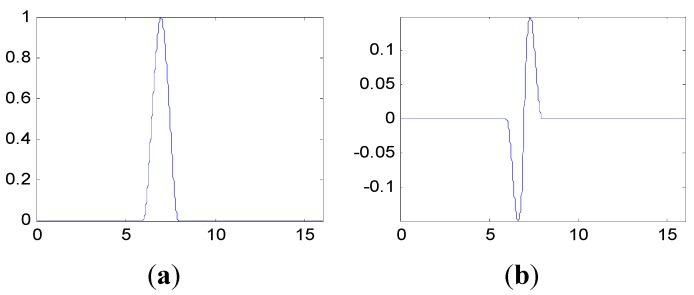
The multiple scaling functions of cubic Hermite splines. (**a**) $\Phi_{1}$; (**b**) $\Phi_{2}$.

**Figure 11:**
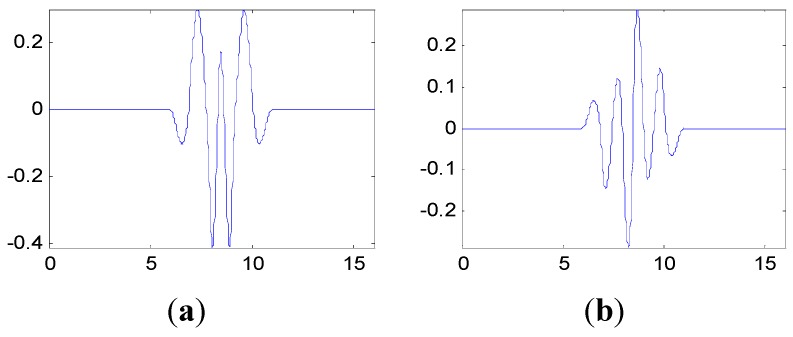
The multiwavelet $\Psi_{1}$ and $\Psi_{2}$ constructed for vibration signal under assembly condition 1. (**a**) $\Psi_{1}$; (**b**) $\Psi_{2}$.

**Figure 12:**
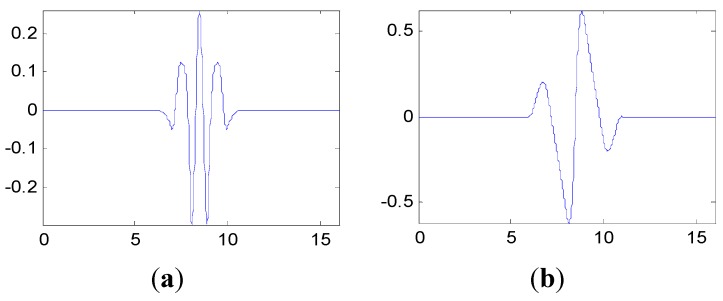
The multiwavelet $\Psi_{1}$ and $\Psi_{2}$ constructed for vibration signal under assembly condition 2. (**a**) $\Psi_{1}$; (**b**) $\Psi_{2}$.

**Figure 13:**
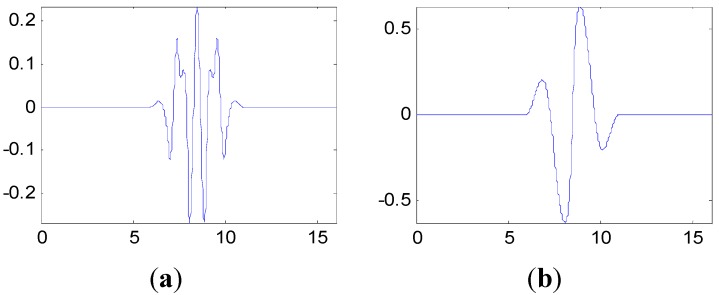
The multiwavelet $\Psi_{1}$ and $\Psi_{2}$ constructed for vibration signal under assembly condition 3. (**a**) $\Psi_{1}$; (**b**) $\Psi_{2}$.

The proposed method in this paper is used to process the measured vibration signal. First, generate the customized multiwavelet basis function, which is lifted from cubic Hermite splines (Strela *et al.* 1994 [29]), as the original multi-scaling function. Due to the simple waveform, more freedom and flexibility can be obtained to construct the multiwavelet with customized properties. The multiple scaling functions are shown in Figure 10. In this and the next special applications on fault detection, the vanishing moment is set as p=4 and the support length is 5, based on the actual requirements. In fact, the vanishing moment and support length can be selected and determined according to the special engineering requirements. After the optimization process based on the genetic algorithms, the customized multiwavelet functions, derived from cubic Hermite splines, are generated for measure vibration signal under the three different assembly conditions displayed in Figure 11, Figure 12 and Figure 13, respectively.

**Figure 14:**
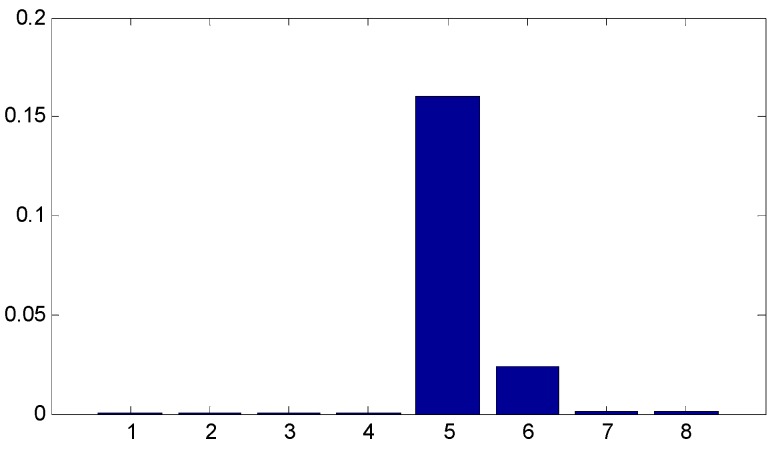
The distribution of the frequency band energy on the ensemble multiwavelet transform coefficients in the assembled quality condition 1.

**Figure 15:**
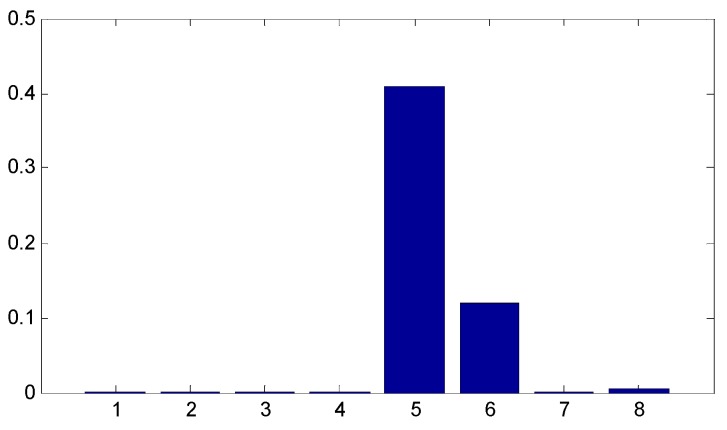
The distribution of the frequency band energy on the ensemble multiwavelet transform coefficients in the assembled quality condition 2.

**Figure 16:**
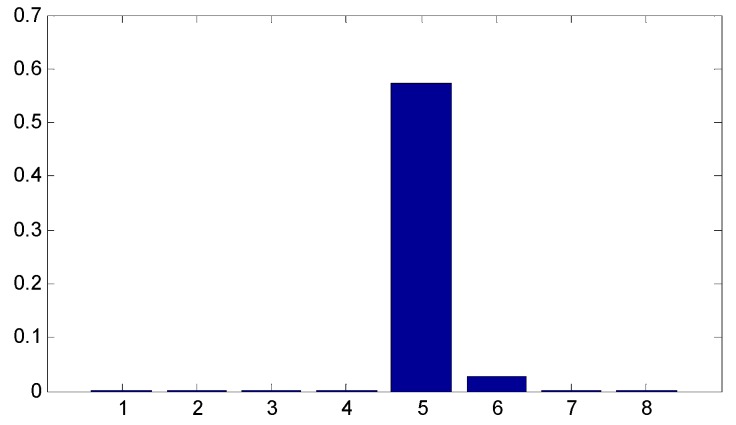
The distribution of the frequency band energy on the ensemble multiwavelet transform coefficients in the assembled quality condition 3.

Since the ensemble multiwavelet analysis method is applied to analyze the measured vibration data of an assembled quality condition to the extent of level 3 by the research experience, there are eight frequency bands attained. Each frequency band has the same bandwidth. According to Equation (18), the frequency band energy of the ensemble multiwavelet transform coefficients from three assembled quality states is calculated and the corresponding distributions are displayed in Figure 14, Figure 15 and Figure 16, respectively. We can find that the frequency band energy of the ensemble multiwavelet transform coefficients from the three different assembled quality stats shows some similarities and differences. The similarities are that the fifth frequency band aggregates the main frequency band energy of the ensemble multiwavelet transform coefficients from three assembled quality conditions, and, meanwhile, the energy in other transform frequency bands are much smaller. The differences are that with the assembled quality from looseness to tightness, the sixth frequency band energy decreases, and, meanwhile, the main frequency band (the fifth frequency band) energy of the ensemble multiwavelet transform coefficients increases. The normalized ensemble multiwavelet information entropy of the acquired four vibration data under each assembled quality states is, respectively, calculated based on Equation (19), which is displayed in Table 1. Compared with the three assembled quality states, the normalized ensemble multiwavelet transform information entropy decreases distinctly with the assembled condition from looseness (fault condition 1) to tightness (normal condition 3). The result shows that the developed method can identify the bolts looseness fault of the dismountable disk-drum aero-engines effectively.

**Table 1:** The normalized ensemble multiwavelet transform information entropy of three assembled conditions.

	Signal 1	Signal 2	Signal 3	Signal 4	Average
Condition 1	0.340	0.328	0.314	0.310	0.323
Condition 2	0.273	0.258	0.241	0.239	0.253
Condition 3	0.167	0.155	0.143	0.147	0.153

### 4.2. Case 2: Analysis on the Vibration Signals from Engineering Field

Maintenance is indispensable in making sure that the components continue to perform the functions for which they are designed. The basic objectives of the maintenance activity are to deploy the minimum resources to ensure system reliability and to ensure that components perform their intended functions properly. At present, the scheduled maintenance, known as time based (or planned) maintenance, and involving repair at regular time intervals, is still widely adopted on most of the aero-engine in service in China. Due to vibrations exceeding limits, the aero-engine of military aircraft in this case cannot be used normally after working for 446 h. However, the first scheduled repair time interval is 500 h. Thus, the reason for the overweight vibration, and reasonable maintenance are needed. According to engineering experience, the fault called rupture accident of the labyrinth seal toothed disk in the high pressure compressor rotor of an aero-engine emerges frequently. In order to solve this problem, our group carried out the related study on the assembly looseness and crack fault condition monitoring of this dismountable disk-drum aero-engine rotor.

**Figure 17:**
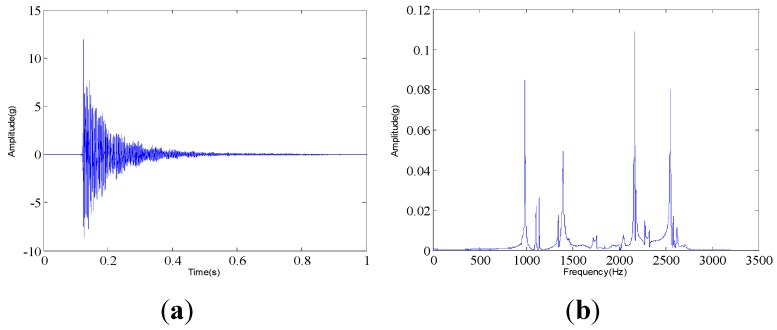
The measured vibration signal of the aero-engine rotor in case 2 and the corresponding frequency spectrum. (**a**) vibration signal; (**b**) frequency spectrum.

The dynamic response signal is acquired from the experimental system, as shown in Figure 6. The sample frequency is 6400 Hz. The dynamic response signal of this aero-engine rotor from one accelerometer and the corresponding frequency spectrum are shown in Figure 17. The dynamic response signal is also damped in the time domain. From the frequency spectrum, the largest amplitude value occurs at 2200 Hz and there are three other main spectral peaks at 1000 Hz, 1400 Hz and 2600 Hz. It is shown that we cannot find the evident features to identify the assembled condition directly, in both time and frequency domains.

**Figure 18:**
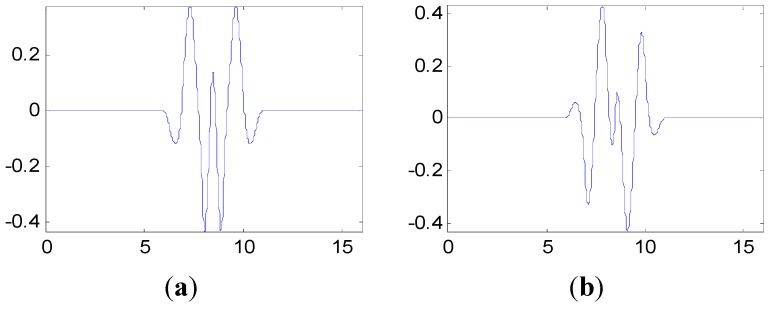
The multiwavelet $\Psi_{1}$ and $\Psi_{2}$ constructed for vibration signal in case 2. (**a**) $\Psi_{1}$; (**b**) $\Psi_{2}$.

**Figure 19:**
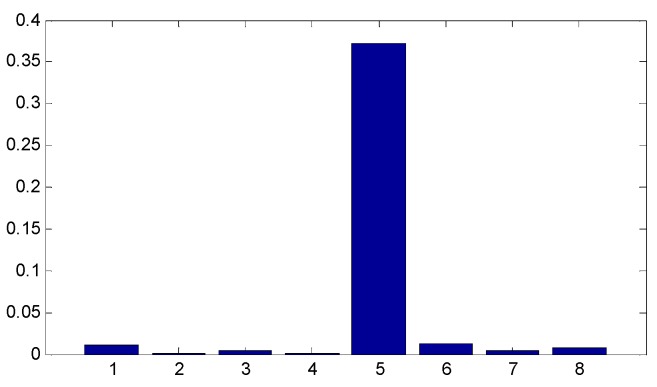
The distribution of the frequency band energy on the aero-engine rotor in case 2.

The proposed method in this paper is applied to the condition monitoring of this aero-engine rotor. The ensemble multiwavelet functions constructed from cubic Hermite splines for the vibration signal in Figure 17 are shown in Figure 18. Since the ensemble multiwavelet transform is applied to process the signals of an assembled quality condition to the extent of level 3, by research experience, there are eight frequency bands attained. According to Equation (18), the frequency band energy of the ensemble multiwavelet transform coefficients is, respectively, computed and the distribution is illustrated in Figure 19. We can find that the main frequency band energy of the ensemble multiwavelet transform coefficients for this aero-engine rotor also lies in the fifth frequency band and the energy in other frequency bands are much smaller, which is similar to that in the experiment in case 1. The differences are that the energies of the first frequency band and the seventh frequency band increase distinctly.

**Figure 20:**
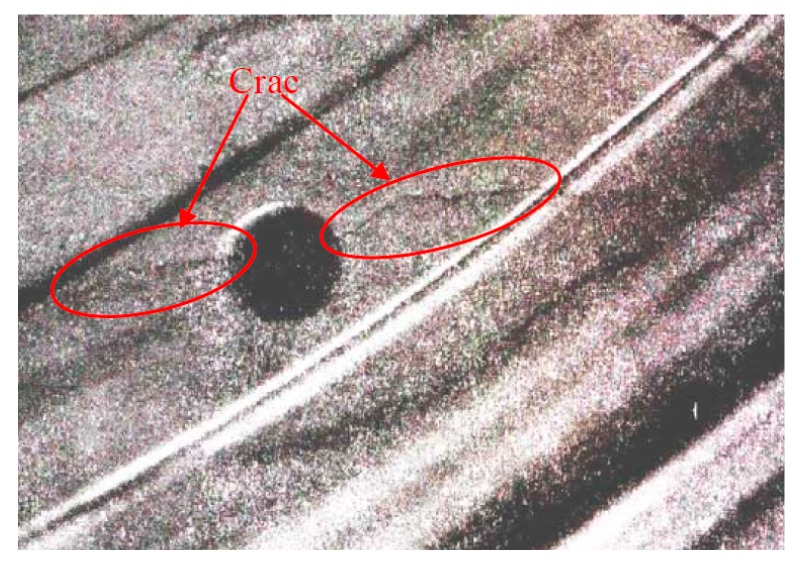
The photograph of this rotor with the crack fault on the pressure hole.

In order to reflect and evaluate the state of this aero-engine rotor, the normalized wavelet information entropy of the acquired signal is computed according to Equation (19), which is shown in Table 2. According to the results, the normalized ensemble multiwavelet transform information entropy of this rotor is a little larger than the value of condition 2 in case 1, which implies that there is an assembly looseness or crack fault in this aero-engine rotor. The faulty rotor is detached for further validation. Through this overhauling, cracks of different degrees on seven pressure holes in the labyrinth seal toothed disk of this rotor are found. The photograph of this rotor with a crack fault on a pressure hole is shown in Figure 20. The result reflects that the assembly looseness fault of the rotor result in this maintenance before the scheduled maintenance. Meanwhile, the result implies that the normalized ensemble multiwavelet transform information entropy possesses excellent performance in the assembly looseness fault detection of the aero-engine rotor, which provides an effective technology for the condition monitoring and health management on the aero-engine rotor.

**Table 2:** The normalized ensemble multiwavelet transform information entropy of the aero-engine rotor in case 2.

	Signal 1	Signal 2	Signal 3	Signal 4	Average
Case 2	0.292	0.277	0.279	0.270	0.280

## 5. Verification on Robustness of the Used Methods against Noise

The effectiveness of the proposed method on fault diagnosis of demountable disk-drum aero-engine rotor has been verified by the two cases in Section 4. Moreover, the robustness of the used methods against noise will be tested and verified in this section by simulation and field experiments.

### 5.1. Simulation Experiment Verification

**Figure 21:**
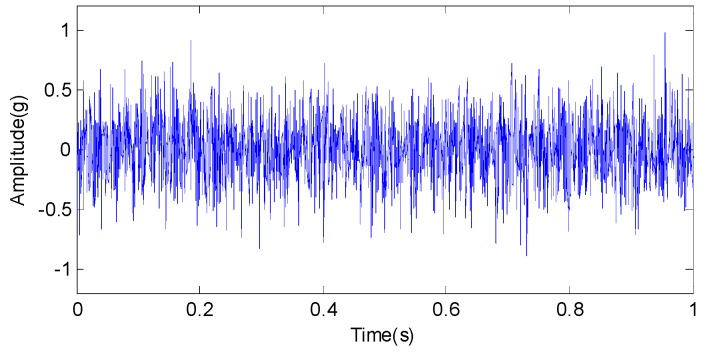
The simulated signal sample with White Gaussian noise.

**Figure 22:**
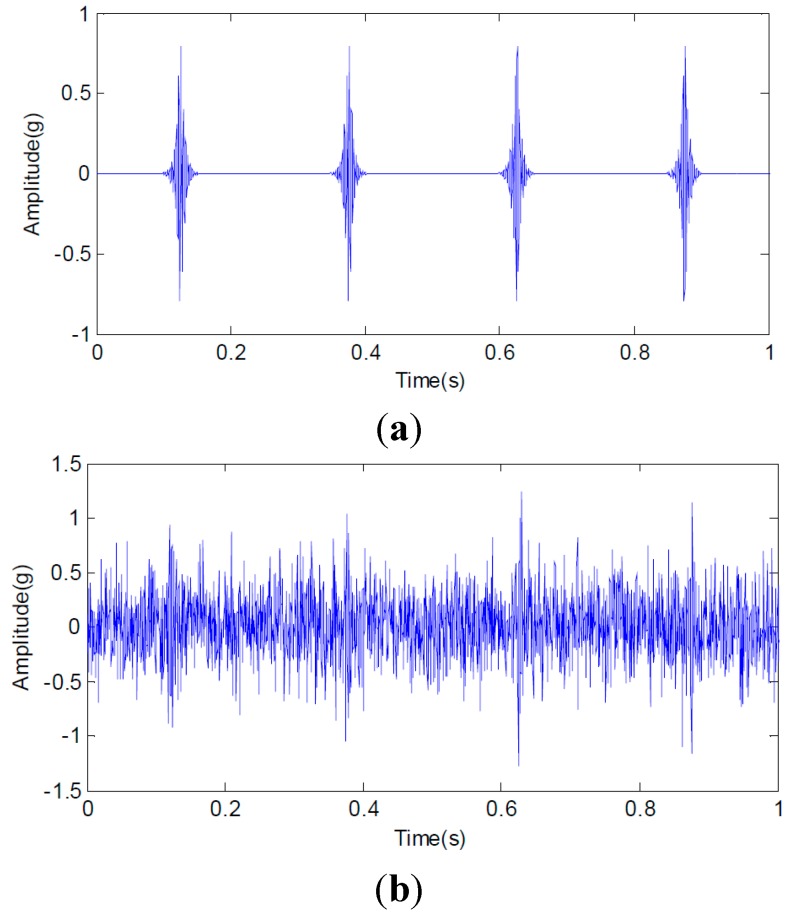
(**a**) The periodic impulse signal sample with a period of 0.25 s and (**b**) the noisy impulse signal sample.

To verify the feasibility of the proposed method for noisy signal analysis, a simulation experiment is designed and carried out. First, 100 simulated signals with White Gaussian noise are generated to simulate the actual case without fault and a sample is shown in Figure 21. When a defect appears on one of the main parts of rotating machinery, such as bearing or gearbox, periodic impulse signals are generated in operation. Nevertheless, influenced by non-stationary operation, noise in sampling and so on, it is very difficult to extract features corresponding to the defect. To simulate the related actual case, 100 simulated signal of the length 2048 is composed for the periodic impulses with a period of 0.25 s, as illustrated in Figure 22a. The sampling frequency is 2048 Hz. White Gaussian noise is added with the signal-to-noise ratio per sample of 9 dB, illustrated in Figure 22b. Obviously, the impulse components are masked by the noise, and we can hardly find the features of periodic impulses of the noisy signal sample in Figure 22b.

**Figure 23:**
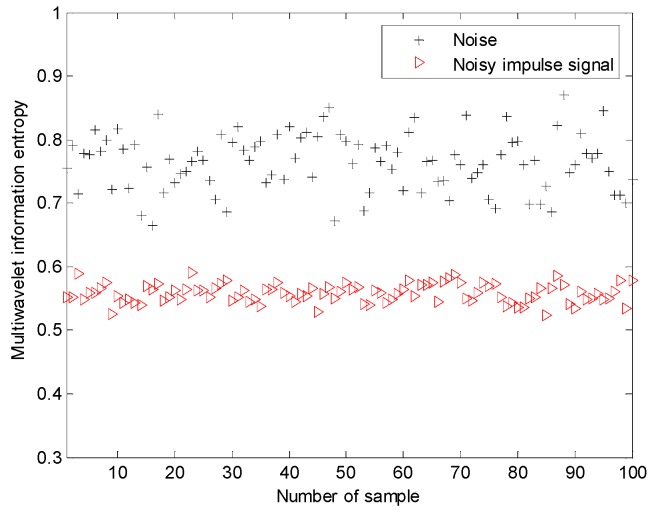
The multiwavelet information entropy values of all the simulated signals.

The proposed multiwavelet method is adopted for analyzing all the simulated signal samples. Since the multiwavelet transform is applied to process the signals of all the simulated condition to the extent of level 3, by research experience, there are eight frequency bands attained. The normalized multiwavelet transform information entropy of the simulated signal is computed according to Equation (19), which is shown in Figure 23. According to Figure 23, we can find that the multiwavelet entropy values of White Gaussian noise samples on normal condition are 0.67–0.83. Meanwhile, the multiwavelet entropy values of noisy simulated signals on fault condition are 0.52–0.59. The result shows that the proposed method can effectively distinguish the different conditions using the noisy signals.

### 5.2. Field Test Experiment Verification

**Figure 24:**
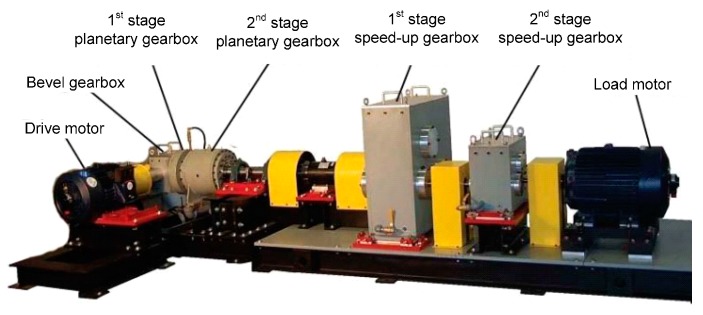
The planetary gearbox test rig.

The planetary gearbox test rig shown in Figure 24 was designed to perform verification experiments for the multiwavelet analysis method on fault diagnosis of planetary gearbox by noisy vibration signals. The test rig includes a 20 HP drive motor, a bevel gearbox, two planetary gearboxes, two speedup gearboxes and a 40 HP load motor. The load was applied through the drive motor. Sensors were installed at the output shaft of the second stage planetary gearbox. There are three planet gears in the first stage planetary gearbox and four planet gears in the second stage planetary gearbox. The first stage sun gear is connected to the bevel gear by shaft #1. The first stage planet gears are mounted on the first stage carrier, which is connected to the second stage sun gear by shaft #2. The second stage carrier is located on shaft #3. Ring gears of the first stage and the second stage planetary gearboxes are mounted on the housing of their stages, respectively.

**Figure 25:**
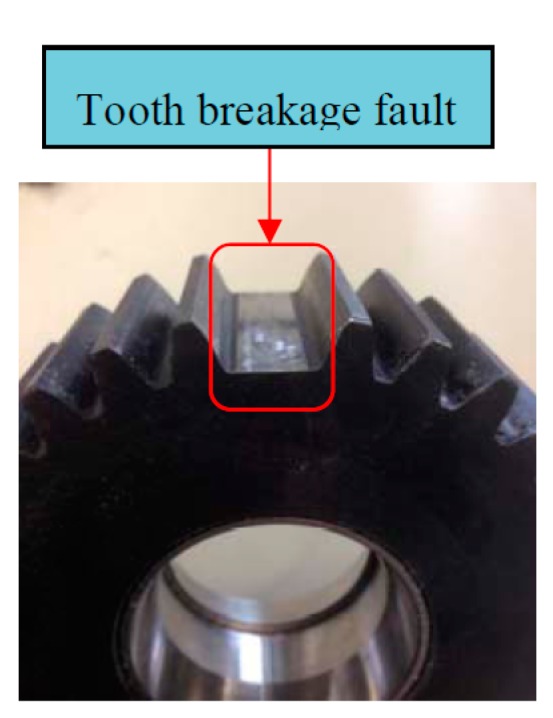
The photograph of tooth breakage fault in planet gear.

**Figure 26:**
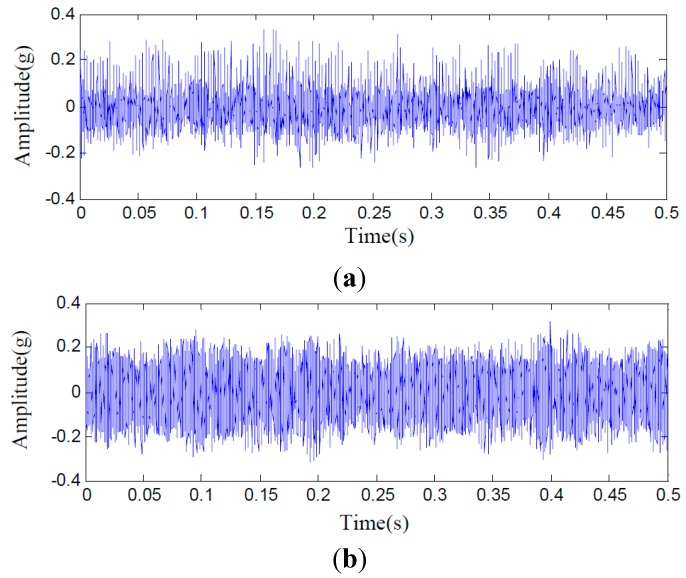
Typical raw data samples of experimental planetary gearbox in normal condition (**a**) and fault condition (**b**).

In this test rig, accelerometer sensors are installed on the casing of the second stage planetary gearbox. The sampling frequency is 10 KHz. A planetary gearbox dataset containing two subsets is obtained from the experimental system under the two different operating conditions. The two conditions include faulty and normal conditions on planetary gearbox. Planet gear in experimental planetary gearbox with tooth breakage fault is shown in Figure 25. Each data subset corresponds to one of the two conditions and it consists of 100 samples. Each sample is a vibration signal containing 5000 sampling points. Figure 26 gives typical raw data samples for each condition. Figure 26a,b, respectively, show the two conditions of planetary gearbox: normal condition and tooth breakage fault in planet gear. From the raw vibration signals, it is not easy to identify the different faults and it is extremely difficult to separate the normal condition and fault conditions using only the raw vibration signals. Thus, in order to identify the two different conditions of planetary gearbox accurately, it is necessary to apply an efficient method.

**Figure 27:**
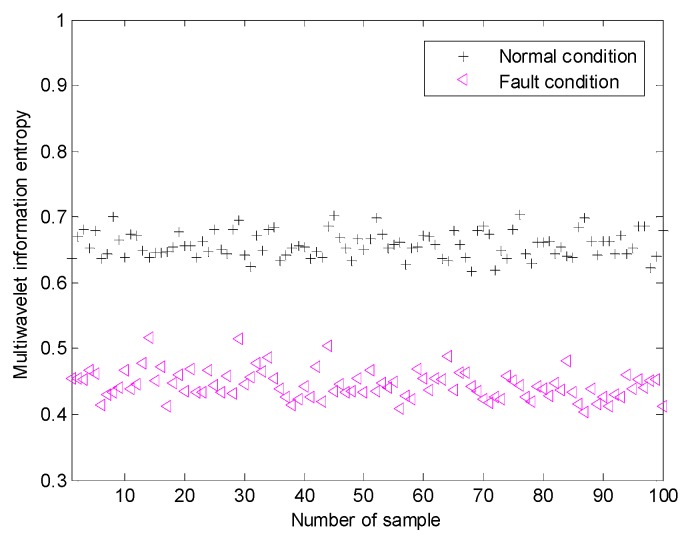
The multiwavelet information entropy values of all the of vibration signals on experimental planetary gearbox.

The proposed method is adopted for this task. The normalized multiwavelet transform information entropy of the condition vibration signals is computed according to Equation (19), which is shown in Figure 27. According to Figure 27, we can find that the multiwavelet entropy values of vibration data on normal condition are 0.62–0.71. Meanwhile, the multiwavelet entropy values of vibration data on fault condition are 0.39–0.52. The result shows that the proposed method can accurately identify the fault condition of planetary gearbox based on noisy condition vibration data.

## 6. Conclusions

A fault diagnosis method based on ensemble multiwavelet analysis is proposed for the condition identification of aero-engine rotor. Multiwavelet has been proven to be a powerful tool to describe the non-stationary vibration signal due to the property of multi-resolution analysis and the multiple wavelet basis functions. To overcome some mentioned limitations, ensemble multiwavelet basis function is constructed via a symmetric multiwavelet lifting scheme. Then, the ensemble multiwavelet transform is performed on the vibration signal of aero-engine rotors. The relative energy in each frequency band of the ensemble multiwavelet transform coefficients, which is equivalent to a percentage of the whole signal energy, is taken as the probability. Normalized information entropy is computed based on the relative energy to reflect and evaluate the state of aero-engine rotor. The proposed method is first applied to the fault detection of an experimental aero-engine rotor. Then the proposed method is used for an engineering application and the results shows that the proposed method performed excellently in this task. Finally, the robustness of the multiwavelet method against noise is also tested and verified by simulation and field experiments.

Moreover, it must be said that although a similar multiwavelet method is also be used for equipment condition identification in reference [13], the severe shortcoming of low adaptivity on the developed multiwavelet basis greatly limits the ability of customized lifting multiwavelet on signal feature extraction. In addition, some findings need to be introduced based on the experimental results. First, fault feature identification and extraction is the fundamental and vital step in this method. Thus, the customized construction of the ensemble multiwavelet basis function is a very key step in this method, which greatly affects the performance of fault detection. Although the multiwavelet lifting scheme is a popular method, we are looking forward to developing some more novel and effective methods for construction. Next, evaluation index is another key factor for the fault detection of aero-engine rotors as well as the construction of an ensemble multiwavelet basis function. The evaluation index must reflect the state information from the fault feature sensitively and effectively. A more useful index should be studied to reflect the state of the development of faults comprehensively in the future. All in all, ensemble multiwavelet analysis method can play a more important role in the detection of faults in aero-engine rotors in the future.

In addition, the authors have attracted some interesting results and will carry out a related study in the future. First, the authors have checked the noise attenuation performance in simulation and experiments. However, it is also of significance to study the noise attenuation ability from the technical/methodological viewpoint by referring to the known model-based and signal-based methods [35]. Second, some interesting techniques, such as distributed sensor networks [36] and fault-tolerant techniques [37], are certainly useful for enhancing the ability of carrying out fault diagnosis of mechanical equipment. Thus, the above-mentioned techniques will be taken into consideration to study with the proposed method in future work.
